# MRI-Based Volumetry Correlates of Autobiographical Memory in Alzheimer's Disease

**DOI:** 10.1371/journal.pone.0046200

**Published:** 2012-10-10

**Authors:** Nathalie Philippi, Vincent Noblet, Anne Botzung, Olivier Després, Félix Renard, Giorgos Sfikas, Benjamin Cretin, Stéphane Kremer, Lilianne Manning, Frédéric Blanc

**Affiliations:** 1 Laboratoire d'Imagerie et de Neurosciences Cognitives (UMR 7237), Université de Strasbourg, Centre National de la Recherche Scientifique, Strasbourg, France; 2 Service de Neurologie - Unité de Neuropsychologie, Centre Hospitalo-Universitaire de Strasbourg, Strasbourg, France; 3 Laboratoire des Sciences de l'Image, de l'Informatique et de la Télédétection (UMR 7005), Université de Strasbourg, Centre National de la Recherche Scientifique, Strasbourg, France; 4 Service de Radiologie, Centre Hospitalo-Universitaire de Strasbourg, Strasbourg, France; University of Cambridge, United Kingdom

## Abstract

The aim of the present volumetric study was to explore the neuro-anatomical correlates of autobiographical memory loss in Alzheimer's patients and healthy elderly, in terms of the delay of retention, with a particular interest in the medial temporal lobe structures. Fifteen patients in early stages of the disease and 11 matched control subjects were included in the study. To assess autobiographical memory and the effect of the retention delay, a modified version of the Crovitz test was used according to five periods of life. Autobiographical memory deficits were correlated to local atrophy via structural MRI using Voxel Based Morphometry. We used a ‘lateralized index’ to compare the relative contribution of hippocampal sub-regions (anterior *vs* posterior, left *vs* right) according to the different periods of life. Our results confirm the involvement of the hippocampus proper in autobiographical memory retrieval for both recent and very remote encoding periods, with larger aspect for the very remote period on the left side. Contrary to the prominent left-sided involvement for the young adulthood period, the implication of the right hippocampus prevails for the more recent periods and decreases with the remotness of the memories, which might be associated with the visuo-spatial processing of the memories. Finally, we suggest the existence of a rostrocaudal gradient depending on the retention duration, with left anterior aspects specifically related to retrieval deficits of remote memories from the young adulthood period, whereas posterior aspects would result of simultaneous encoding and/or consolidation and retrieval deficit of more recent memories.

## Introduction

Autobiographical memory, in its episodic component (AbM), refers to the ability to remember personal events with a unique and detailed spatial and temporal context that a person is able to re-experience, as opposed to personal semantic memory (PS) which refers to general knowledge with personal implication. It is widely agreed that the hippocampus and other related medial temporal lobe (MTL) structures play a central role in the formation as well as the consolidation of newly learned material [1], [2], [3]. By contrast, the implication of MTL in episodic memory retrieval mechanisms remains controversial, partially due to difficulties in assessing episodic autobiographical memory. Retrieving episodic autobiographical memories (AbMs) entails a complex generative retrieval process, episodic memory traces, semantic knowledge and visuo-spatial imagery [4], which requires sophisticated assessment tools.

### 1.1. Neural substrates of AbM retrieval in healthy individuals

The neural substrates of AbM retrieval have been extensively explored in functional neuroimaging in healthy subjects (e.g. [5],[6],[7],[8],[9]). These studies have revealed a complex cerebral network, showing several variations as a function of the paradigm employed (e.g. blocked *vs*. event-related) and shared regions that are referred to as the ‘core’ network (see [10] for a review). Among them, the ventrolateral prefrontal cortex (BA 47) is thought to be involved in retrieval strategies and reconstructive mnemonic processes, while the medial prefrontal cortex (BA 10) would be crucial for self-referential processing. Furthermore the lateral temporal lobes, particularly the middle temporal gyrus (BA 21) are associated with the semantic component, i.e. general autobiographical knowledge associated to personal events. Finally, the temporo-parietal junction, the retrosplenial and the posterior cingulate cortices, are thought to support the visuo-spatial context processing of events, whereas other posterior cortical regions (i.e., occipital and parietal cortices), although less frequently reported, are generally associated with visual imagery. It is well established in AbM literature that the MTL plays an important role in AbMs retrieval, however, its implication remains controversial regarding the delay of retention (see [10] for a review). Thus, the standard theory of consolidation (STC ; [3]) suggests that declarative memory representations within the neocortex become stable and independent from MTL structures over time, while the multiple trace theory (MTT ; [2], [11]) puts forward that episodic memory retrieval relies on the MTL independently of the age of the memories (see also the alternative ‘transformation’ theory [12]). A majority of functional neuroimaging studies in healthy subjects have found that MTL structures are activated in response to both recent and remote episodic events [5], [6], [8], [13], [14], [15], [16], [17], [18], [19], while others found greater activation for this region with recent rather than remote events [20], [21], [22]. This first group of studies is in line with the MTT, whereas the second is in accord with the STC. These inconsistencies may result from differences between the study designs: type of material, time course of the recollective experience, subjects' age, and the recollective qualities of AbMs such as emotion, richness in details, vividness, and personal significance. An alternative approach to functional imaging in healthy subjects is to take advantage of a condition such as Alzheimer's disease (AD), that triggers both MTL volume loss and episodic retrograde memory deficits, and to correlate behavioral performances to the extent of local atrophy using a volumetric analysis.

### 1.2. AbM studies in AD

Patients with AD provide a unique opportunity to study the relationship between AbM retrieval deficit and MTL atrophy, which occurs early on over the course of the disease and appears well correlated to the pathological processes [23]. Numerous studies have revealed an impairment of AbM in AD [24], [25], [26], [27], [28], [29], [30], [31], [32], [33], [34]. The majority of these studies have shown temporally graded amnesia, though some [24], [30], [34] have suggested that the impairment was independent of the period of life when considering strictly episodic autobiographical memory. Moreover, other studies have demonstrated a correlation between retrograde memory and anterograde memory scores, suggesting the implication of the MTL in AbM retrieval, in keeping with previous results [24], [35]. To address this issue, Eustache et al. [36] investigated the status of autobiographical memory in AD compared to resting state PET. The authors found significant positive correlations between AbM scores and hippocampal hypometabolism for recent memories only, whereas remote memories where correlated to hypometabolism in the left prefrontal regions. As remote memories corresponded to generic rather than strictly episodic events, the lack of hippocampal involvement could reflect a generalization (or ‘semanticization’) of initially episodic AbMs rather than the disengagement of the hippocampus in retrieval. Hence, the author concluded that the results were compatible with both theories of memory consolidation. To the best of our knowledge, only one study has investigated the relationship between AbM deficit and MTL atrophy in a volumetric study using a multivariate analysis method of partial least squares [35]. The authors confirmed the dissociation between the episodic and semantic component of autobiographical memory, with PS scores correlated to external temporal lobe volume, while AbM scores were correlated to MTL. This was the case independent of the period of life, in keeping with the predictions of the MTT. However, the results reflected the implication of the MTL rather than the hippocampus proper.

### 1.3. Objectives

The objective of the present study was to investigate the cerebral network sustaining AbM in patients in early stages of AD, using voxel based morphometry (VBM). A group of AD patients and matched healthy elderly subjects were assessed for AbM using the modified Crovitz test (MCT) and underwent a 3D T1 MRI sequence. Our hypothesis was that we would be able to identify some of the regions described in functional neuroimaging in healthy subjects, within those atrophied in AD patients. Among those regions, we expected the involvement of the MTL and the hippocampus proper independent of the age of the memories, according to the MTT.

## Materials and Methods

### 2.1. Participants

Fifteen AD patients, aged 69 to 84 years old, right-handed, native French-speakers, were recruited through the Neuropsychology Unit, Service of Neurology, University Hospital of Strasbourg, France. A diagnosis of probable AD was made according to the criteria of the NINCDS-ADRDA [37] and to the more recent criteria proposed by Dubois et al. [38], whose specificity has recently been proved to be reliable (i.e. 93%, see [39]). Every patient disclosed a history of progressive cognitive impairment and underwent a battery of neuropsychological tests as part of their follow-up, confirming episodic memory impairment with no cueing-related improvement (Free and Cued Selective Reminding Test [40]), either isolated or associated with other cognitive changes (visuospatial abilities, praxia, language…). The stage of the disease was prodromal in 8 patients, according to the criteria proposed by Dubois et al. [38] with preserved social functioning and instrumental activities of daily living, with a Clinical Dementia Rating score of 0.5 (CDR [41]). The seven remaining patients had mild dementia (CDR = 1). The patients' mean score on the MMSE was 24.1 (SD 3.0). The patients were further assessed using the Verbal Paired Associates test [42] to determine anterograde verbal memory and had a mean score of 8.7 (SD 4.2). Every patient had MRI evidence of MTL atrophy, as assessed visually by a senior neurologist (FB [43]), and eight of them underwent SPECT examination, which revealed a typical pattern of reduced perfusion in the MTL and parietal regions. The eight ‘prodromal’ AD patients also underwent a cerebrospinal fluid (CSF) biomarkers examination and displayed abnormal results with a combination of low amyloïdβ1–42 concentrations (<500 ng/L), increased total tau concentrations (>500 ng/L), increased phospho-tau concentrations (>60 ng/L). We excluded patients who disclosed a history of major depression or neurovascular disease, abnormal physical neurological examination or other possible causes of dementia, such as Lewy body dementia. The patients were under medication comprised of one or two AD-specific drugs (acetylcholine-esterase inhibitor or acetylcholine esterase inhibitor and memantine).

Eleven healthy elderly subjects matched for sex ratio, age distribution, education level and handedness were tested. For inclusion, subjects had to be free of major depression, central neurological disease, or cognitive complaint, and without abnormal neurological examinations. The control subjects underwent the Verbal Paired Associates test [42] in addition to the remote memory tests, with normal scores (mean 18.0/21 and SD 1.7).

Tab. 1 provides demographic details of the two groups. Inter-group comparisons were made using Mann-Whitney U pair tests for quantitative characteristics (age distribution and education level), and qualitative characteristics (gender and handedness) were analyzed using χ^2^ tests. These analyses revealed that the groups were well matched for both.

**Table 1:** Comparison of the general data for the two groups.

	N	Sex ratio (F/M)	Mean age (SD) in years	Mean education level (SD) in years	Hand dominance ratio (L/R)
**Control group**	11	3/8	73,3 (4,80)	13,1 (2,55)	0/11
**Patient group**	15	3/12	76,7 (5,62)	11,9 (2,46)	0/15
**Statistical analysis**	-	Khi^2^ = 0,19	U [26] = 48,5	U [26] = 64	-
	-	*p* = 0,66	*p* = 0,08	*p* = 0,34	-

F: female; M: male; L: left; R: right; SD: standard deviation.

#### Ethics statement

All participants provided informed written consent for the study according to the Declaration of Helsinki and the MRI procedure was approved by the local Ethics committee of East France (IV).

### 2.2. Autobiographical memory assessment

A French version [44] of the MCT [26] was used to assess episodic AbM. Participants were asked to produce detailed and specific recollections in response to 6 specific cues (e.g. letter, train, surprise…), each prompted five times in order to elicit memories from five periods of life (0–9 years, 10–29 years, 30–59 years, 60 to current age minus 1 year – referred to as ‘after 60 years’-, and previous year; e.g. “Could you recall an event that happened in relation to *a train* before *you turned 9*"). Presentation of the words and time periods were randomized and no time-limit was set. A second cue was given for each possible response before considering that a participant had no memory. The participants were encouraged to recall as many details as possible (spatial, temporal, and emotional) and incomplete responses were probed with further questions (e.g. “Tell me more."; “Do you remember where it took place?"). Memories were subsequently scored on a 5-point scale, and fell in five different categories, as follows: 0, absence of response; 1, semantic facts related to the target word; 2, poorly detailed generic or repeated events; 3, detailed generic or repeated events; 4, poorly detailed specific events; 5, richly detailed specific events. Thirty memories were thus prompted for recollection, with a maximal possible raw score of 150.

### 2.3. Statistical analyses

Concerning the autobiographical assessment, ANCOVAs were performed to compare the raw scores obtained in the MCT as a function of the different periods of life, including age as a nuisance covariate. Post-hoc analyses were performed with the Fisher's LSD test.

### 2.4. Voxel based morphometry analyses

Voxel-based morphometry (VBM) is a technique that identifies cerebral volume changes on a voxel-by-voxel basis from structural MRI data. It provides the opportunity to correlate performance on behavioral measures of interest to regional cerebral volume. We used VBM to investigate the neuroanatomical correlates of AbM deficit to regions of gray matter atrophy in both AD and control groups. In order to map the regions related to the AbM deficit revealed in our patients (see results), we correlated the gray matter (GM) volume at a voxel level with the MCT scores in both patient population and control group.

Each participant underwent a high resolution MRI scan, within six months from neuropsychological testing. High resolution anatomical images were obtained on a General Electric SIGNA HDx MR 3T MRI (Milwaukee, USA) using a Fast Spoiled Gradient Echo sequence (TR = 7.2 ms, TE = 2.3 ms, flip angle = 20°, FOV = 22 cm, matrix = 256×256, 176 slices of 1 mm).

Voxel-based morphometry analyses included image pre-processing and statistical analyses. These steps were carried out using the SPM8 software package (Wellcome Department of Imaging Neuroscience, London; http://www.fil.ion.ucl.ac.uk/spm) running on Matlab R2010a (MathWorks, Natick, MA). Anatomical MRI images were spatially pre-processed using standard procedures [45]. All T1 structural images were first segmented, bias corrected and spatially normalized to the Montreal Neurological Institute (MNI) space using the unified segmentation procedure [46]. Then the DARTEL registration toolbox was used to build a study-specific template and to bring into alignment all the segmentation images. The VBM analysis was done on modulated gray matter images, whereby the gray matter value in each voxel is multiplied by the Jacobian determinant derived from the spatial normalization. This procedure preserves the total amount of gray matter from the original images. These modulated gray matter images were smoothed with a Gaussian kernel (FWHM: 12 mm).

Statistical correlations between local gray matter volume and scores on the MCT were then investigated using the General Linear Model (GLM). For the correlation analysis, the smoothed gray matter images of both AD patients and control subjects were entered in the statistical model. Raw scores on the MCT (for the whole lifespan and independently for each period of life, i.e., ‘0–9 years’, ‘10–29 years’, ‘30–59 years’, ‘after 60 years’, and ‘previous year’) were tested successively by entering each of them as a covariate of interest. The correlations were tested using t-contrasts, assuming that decreased AbM would be associated with decreased gray matter volumes. We pooled both AD patients and control subjects in the correlation analysis, which enabled us to consider a wider range of scores on the MCT and to increase the statistical power of our tests (by increasing the number of samples). Considering the group placement (i.e., AD patients or control subjects) as a nuisance variable, we hypothesized that part of the atrophy is explained by normal ageing, on the one hand, and that part of it results from the pathology, on the other hand. Other nuisance covariates were also considered in the model: the age of the subjects, intracranial volume, and gender. As no correlations were found after correction for multiple comparisons (neither with SPM family-wise error – FWE, nor with FDR), a more liberal statistical threshold of *p*<0.005, uncorrected, was considered with a cluster spatial extend of 50 voxels. MNI coordinates were transformed in Talairach coordinates with Xjview (http://www.alivelearn.net/xjview8/). This software allowed us to identify the brain regions and determine the number of voxel within region included in each cluster. In the present work, we refer to the hippocampus according to AAL atlas, as the hippocampus proper plus the dentate gyrus and uncus (http://www.cyceron.fr/upload/editeur/Tzourio_NI_15-273_02.pdf). Reference to the ‘MTL’ includes additionally the entorhinal, perirhinal and parahippocampal cortices [47].

To investigate the prevailing implication of hippocampal subregions with respect to the period of life, we resort to the lateralization index (LI) that is commonly used to quantify the asymmetry of brain activation in functional studies [48]. This was done using an SPM toolbox called LI-toolbox (http://www.medizin.uni-tuebingen.de/kinder/en/research/neuroimaging/software/). The LI index is computed for a given statistical threshold *t* as follows: *LI_(L-R)_ (t)* = (*T_left_ (t)−T_right_ (t)*)/(*T_left_ (t)+T_right_ (t)*) with *T_left_ (t)* (resp. *T_right_ (t)*) the mean of the statistical map thresholded at a significance level *t* in the left (resp. right) region of interest. This index ranges between −1 (right detection only) and 1 (left detection only) and is used in the context of our study to compare left *vs* right hippocampal subregions. In a similar way, we extend this index to compare the involvement of anterior *vs* posterior parts of each hippocampus using the following formulation: *LI_(A-P)_ (t)* = (*T_anterior_ (t)−T_posterior_ (t)*)/(*T_anterior_ (t)+T_posterior_ (t)*) with *T_anterior_ (t)* (resp. *T_posterior_ (t)*) the mean of the statistical map thresholded at a significance level *t* in the anterior (resp. posterior) part of the hippocampus. Since these indices can be very dependent on the chosen statistical threshold *t*, we resort to LI curves (*i.e.*, LI values computed for a range of twenty statistical thresholds) that enable us to better assess the trend towards laterality (or antero-posterior discrimination) in the data. Curves that go through zero tend to invalidate these hypotheses. The information carried out by these curves are summed up in the sequel by their median, median absolute deviation (MAD), minimum and maximum values. For the hippocampal sub-region analysis, we used the AAL atlas. To define the anterior and posterior parts of hippocampus, we determined the coronal plane that divide the hippocampus in two regions of equal volume (*y* = −20 plane according to Talairach coordinates).

## Results

### 3.1. Autobiographical memory assessment

**Figure 1:**
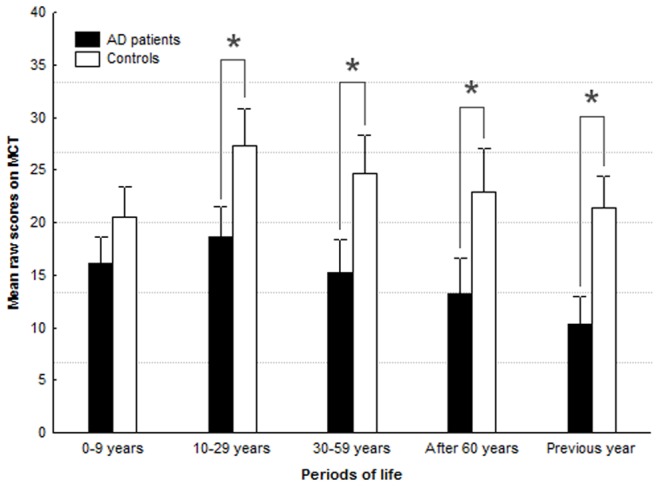
Scores on the MCT for the two groups are shown for the five periods of life.

The mean score obtained on the MCT was significantly inferior in the AD group (71.6, SD 29.0) than in the control group (119.3, SD 8.7), (F_[1, 23]_ = 21.52 ; *p*<.001). Fig. 1 shows the mean scores on the MCT in both groups for the five periods of life.

ANCOVAs showed no significant period effect (F_[4,92]_ = .84; *p* = .50) but a significant interaction between the group factor and life period parameter (F_[4,92]_ = 3.16 ; *p* = .02). Fisher's LSD test for inter-group comparison indicated that AD patients had a significantly lower score than controls for all periods of life (*p* = .03 for ‘10–29 years’; *p* = .02 for ‘30–59 years’; *p* = .01 for ‘after 60 years’; *p*<.01 for the ‘last year’ period) except the ‘0 to 9 years’ period (*p* = .19).

The intra-group comparison showed that the AD patients had significantly higher scores for the ‘10–29 years’ period than any other period (*p* = .04 with the ‘0–9 years’ period, *p* = .01 with the ‘30 –59 years’ period and *p*<.001 for the ‘after 60 years’ and ‘previous year’ periods). Conversely, the scores for ‘recent year’ were significantly lower than for any other period (*p* = .02 with the ‘after 60 years’ period, *p*<.001 for the ‘30–59 years’, ‘10–29 years’ and ‘0–9 years’ periods). The scores for the ‘after 60 years’ period were significantly lower than for the two first period (*p* = .02 for the ‘0–9 years’ period, *p*<.001 for the ‘10–29 years’ period) whereas there was no significant difference in comparison to the ‘30–59 years’ period (*p* = .06).

The intra-group comparison showed that the controls had significantly lower scores for the ‘0–9 years’ period than the two following periods (*p*<.001 with the ‘10–29 years’ period and *p* = .01 with the ‘30–59 years’ period), and the scores for the ‘10–29 years’ period were not only higher than for the first period, but also higher than the two last periods (*p* = .005 for the ‘after 60 years’ period and *p*<.001 for the ‘previous year’). Other comparisons did not reveal significant differences between the scores.

### 3.2. Voxel based morphometry analyses

#### 3.2.1. AbM score on the MCT

**Figure 2:**
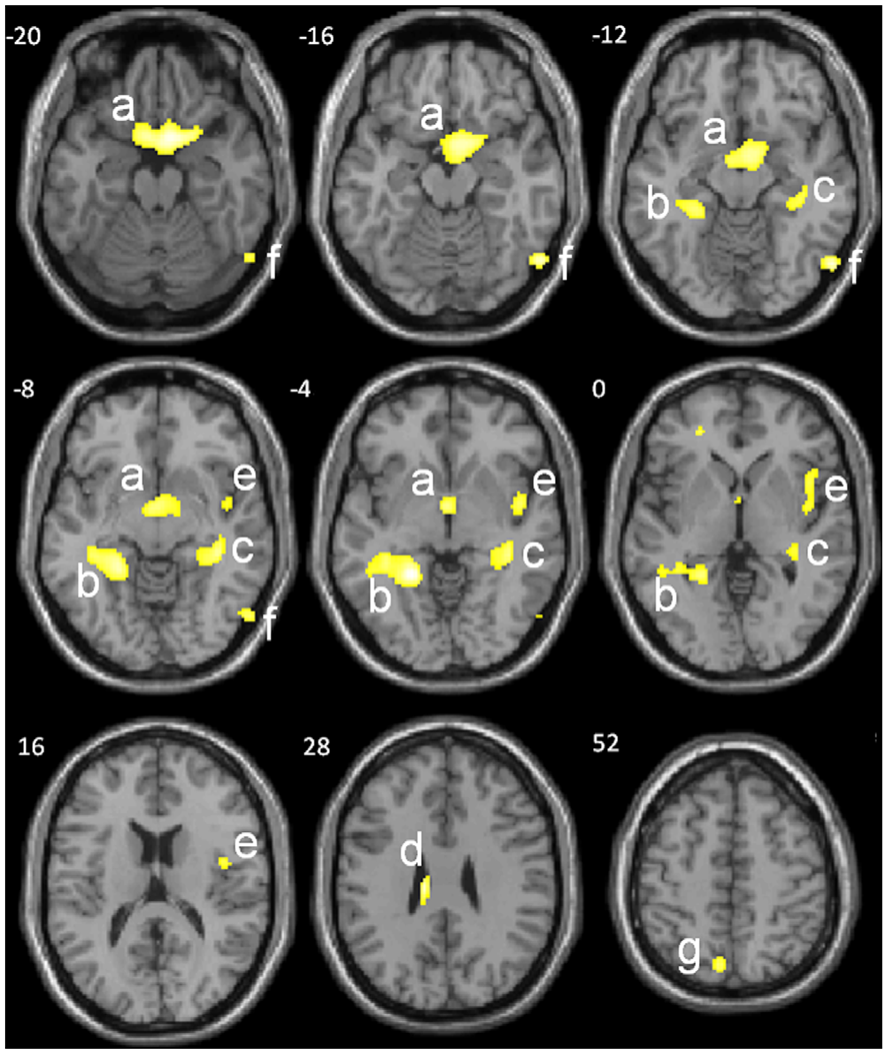
VBM analyses showing brain areas of regional atrophy that correlate with the AbM deficit according to the score on the MCT (*p*<0.005, uncorrected, *k* = 50 voxels, including group, age, intracranial volume, and gender as nuisance covariates), with clusters including (a) left and right ventromedial, ventrolateral, orbial prefrontal cortex and anterior cingulate (BA 11/25/47), (b) left hippocampus (*y* = −30 to *y* = −40), parahippocampus (BA 36/37), retrospenial cortex (BA 30), (c) right hippocampus (*y* = −22 to *y* = −40), (d) left cingulate gyrus, (e) right insula (BA13) and superior temporal gyrus (BA22), (f) right middle occipital gyrus (BA19), (e) left precuneus (BA7). Talairach *z* coordinates are indicated for each slice.

The VBM correlation analysis (*p*<.005, uncorrected, minimal cluster size *k* = 50), pooling the two groups and including intracranial volume, gender, age, and group taken as nuisance covariates, revealed a significant positive correlation between AbM performance and GM volume regions. Seven clusters of atrophy were found (see Fig. 2), whose characteristics are displayed in Tab. 2: the largest, sized 1191 voxels, involved ventromedial prefrontal region extending from the anterior cingulate gyrus to the ventrolateral prefrontal gyrus bilaterally and including BA 11/47/25; the second cluster, sized 630 voxels, involved the hippocampus (*y* = −30 to y = −38), parahippocampal structures (BA 36/37) and retrosplenial cortex (BA 30) on the left; the third cluster, sized 279 voxels, involved the hippocampus (*y* = −28 to *y* = −36), parahippocampal structures (BA 36/37). The four other clusters included respectively right insula (BA13) and superior temporal gyrus (BA22), right middle occipital gyrus (BA19), left precuneus (BA7), and left cingular gyrus. None of the voxels showed significant correlation between the score on the MCT and the GM volume at a threshold of *p*<0.05, and corrected for multiple comparisons (neither with SPM family-wise error – FWE, nor with FDR).

**Table 2:** GM volume regions positively correlated to the score on the MCT in VBM including group, age, intracranial volume and gender as nuisance covariate, with a minimal cluster size (*k*) of 50 voxels and a threshold of *p*<.005, uncorrected.

Brain region	Side	BA	*k*	*x*	*y*	*z*	T-value
**Medial frontal g.**	L	25	1191	−6	10	−20	3.66
**Inferior frontal g.**	L	47	1191	−14	12	−24	3,52
**Rectus g.**	L	11	1191	−6	12	−24	3,83
**Medial frontal g.**	R	25	1191	6	10	−20	3,87
**Inferior frontal g.**	R	47	1191	22	12	−20	3,47
**Rectus g.**	R	11	1191	6	12	−24	3,39
**Anterior cingulate**	L	25	1191	−2	0	−6	3,19
**Anterior cingulate**	R	25	1191	2	2	−10	3,15
**Cingulate gyrus**	L	NA	73	−12	−22	28	3.75
**Insula**	R	13	168	44	0	−4	3.25
**Superior temporal g.**	R	22	168	46	−6	−6	2.96
**Hippocampus**	L	NA	630	−30	−36	−8	3,42
**Parahippocampal g.**	L	36	630	−28	−38	−12	3,24
**Parahippocampal g.**	L	37	630	−30	−38	−10	3,36
**Retrosplenial cortex**	L	30	630	−18	−42	−6	3,24
**Hippocampus**	R	NA	279	36	−32	−8	3,39
**Parahippocampal g.**	R	36	279	34	−38	−6	3.26
**Middle occipital g.**	R	19	144	56	−72	−10	3.50
**Precuneus**	L	7	52	−8	−72	54	2.85

g.: gyrus; L: left; R: right; *x, y, z*: Talairach coordinates; BA: Brodman Area.

#### 3.2.2. AbM scores on the MCT independently for each period of life

**Figure 3:**
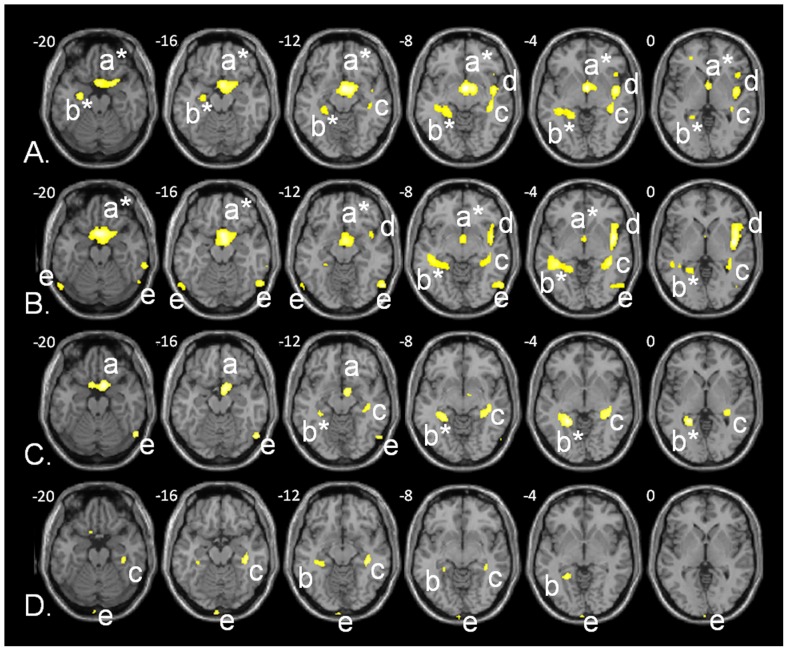
VBM analyses showing brain areas of regional atrophy that correlate with the AbM deficit according to the score on the MCT ( *p*<.005, uncorrected, *k* = 50 voxels, including group, age, intracranial volume, and gender as nuisance covariates), independently for each period of life: (A) for the ‘10–29 years’ period, (B) for the ‘30–59 years’ period, (C) for the ‘after 60 years’ period, and (D) for the “previous year". The clusters include (a) ventro-medial, ventro-lateral, orbial prefrontal cortex +/− anterior cingulate*, (b) left hippocampus +/− parahippocampus, retrosplenial cortex and/or amygdala*, (c) right hippocampus, (d) insula and temporal neocortex, (e) occipital neocortex (precuneus not shown). Talairach *z* coordinates are indicated for each slice.

**Figure 4:**
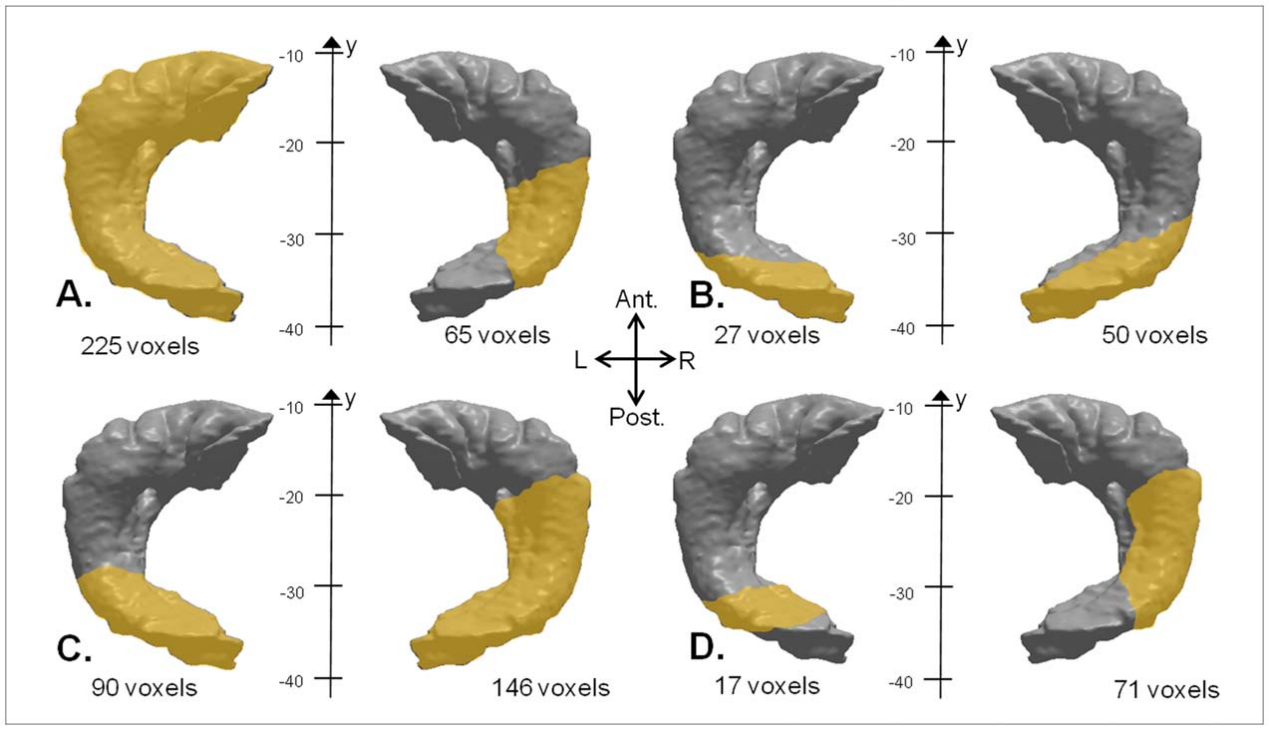
Schematic recapitulation of the hippocampal aspects according to the period of life (*p*<.005, uncorrected, *k* = 50 voxels, including group, age, intracranial volume, and gender as nuisance covariates), including bilateral hippocampal engagement (A) for the ‘10–29 years’ period, (B) for the ‘30–59 years period, (C) for the ‘after 60 years’ period, and (D) for the ‘recent year’. Volumes and Talairach *y* coordinates are indicated for each period and side (L = left, R = right; Ant. = anterior, Post. = posterior).

The VBM correlation analyses (*p*<.005, uncorrected, minimal cluster size *k* = 50 voxels), pooling the two groups and including intracranial volume, gender, age, and group taken as nuisance covariates, revealed a significant positive correlation between AbM performance and GM volume regions, for every period of life except the more recent one (‘0–9 years’). The clusters are shown in Fig. 3 and their characteristics summarized in Tab. 3. Of particular interest, hippocampal involvement existed bilaterally for each of these periods with larger aspects for the ‘10–29 years’ period on the left side and for the ‘after 60 years’ period on the right side (See Fig. 4 for a schematic view summarizing the hippocampal aspects).

**Table 3:** GM volume regions positively correlated to the global score on the MCT and independently for each life-period (‘0–9 years’, ‘10–29 years’, ‘30–59 years’, ‘after 60 years’, and ‘previous year’) in VBM including group, age, intracranial volume, and gender as nuisance covariates, with a minimal cluster size (*k*) of 50 voxels and a threshold of *p*<.005, uncorrected.

Lobe	Frontal lobe				Limbic lobe							Temporal lobe				Parietal lobe		Occipital lobe		Insula
Region/gyrus	Orb.	VL		Med.	Cingulate		Parahipp.		Hippocampus		Amyg.	Sup.	Mid.	Inf.	Fus.	Prc	Psc	II	III	
BA	11	44	47	25	25	30	36	37	Y-Left (V)	Y-Right (V)		22	21	20	37	7	42/43	18	19	13
**Global**	L/R	-	L/R	L/R	L/R	L	L/R	L	−30 to −40 (100)	−22 to −40 (122)	-	R	-	-	-	L	-	-	R	R
**0–9 years**	-	-	-	-	-	-	-	-	-	-	-	-	-	-	-	-	-	-	-	-
**10–29 years**	L/R	-	L/R	L/R	L/R	L	L	L	−10 to −20 (117)−24 to −40 (108)	−22 to −36 (65)	L	R	R	-	-	-	-	-	-	R
**30–59 years**	L/R	R	R	L/R	L/R	L	L	L	−32 to −40 (27)	−28 to −40 (50)	-	-	L/R	R	R	L/R	L	-	L/R	R
**>60 years**	L/R	-	R	L/R	-	-	L	-	−30 to −40 (90)	−20 to −40 (146)	-	-	-	-	R	-	-	-	R	-
<1 year *	L	R	L	L	-	-	-	-	-30 to -34 (17)	−18 to −36 (71)	-	-	-	-	-	-	-	L/R	-	-

The regions are indicated with their side (L: left; R: right) within their belonging lobe, gyrus or region and Brodman area (BA, if applicable) for the global score on the MCT and independently for each period of life. Talairach *y* coordinates (Y) and volumes (in voxels, V) are indicated on both sides for the hippocampal involvement. Orb.: orbito-frontal; VL: ventro-lateral; Med.: medial; Parahipp.: parahippocampal gyrus; Amyg.: amygdala; Sup.: superior temporal gyrus; Mid.: middle temporal gyrus; Inf.: inferior temporal gyrus; Fus.: fusiform gyrus; Prc : precuneus; Psc : postcentral gyrus; II: cuneus or lingual gyrus; III: middle occipital gyrus.

#### 3.2.3. Lateralization index (LI) of hippocampal sub-regions

Testing the involvement of hippocampal sub-regions found in the VBM analyses depending on the period of life (see Fig. 4), LI analyses indicated that the left anterior part of the hippocampus was involved more strongly than the posterior part on the left (median 0.02, MAD 0.00, limit values: 0.00 to 0.68) and more than the anterior part on the right (median 0.16, MAD 0.01, limit values: 0.15 to 0.79) for the ‘10–29 years’ period. Additionally, the posterior part of the left hippocampus was also more strongly involved than on the right (median 0.10, MAD 0.04, limit values: 0.06 to 0.65). On the contrary, for the two following periods, LI analyses showed that the posterior part of the right hippocampus was involved more strongly than the posterior part on the left (median −0.12, MAD 0.04, limit values −0.30 to −0.06 for the ‘30–59 years’ period; median −0.05, MAD 0.00, limit values−0.32 to −0.04 for the ‘after 60 years’ period) and confirmed that it was more strongly involved than the anterior part on the right side (median −0.47, MAD 0.26, limit values: −0.81 to −0.20 for the period ‘30–59 years’; median −0.22, MAD 0.03, limit values −0.95 to −0.20 for the ‘after 60 years’ period). Concerning the ‘recent year’, LI analyses were not conclusive when comparing the implication of the posterior part on the one side and another (median 0.06, MAD 0.01, limit values: −0.45 to 0.12). Overall, the right hippocampus was more strongly involved in its posterior part, not only for the two intermediate periods (cf supra), but also for the ‘10–29 years’ period (median −0.07, MAD 0.04, limit values:−0.63 to 0.25) and ‘recent year’ (median −0.21, SD 0.10, limit values: −0.87 to −0.11).

## Discussion

We examined the cerebral structures implicated in the retrieval of autobiographical memories (AbMs) according to five periods of life with a VBM method enabling us to correlate the AbM deficit and regions of focal atrophy in AD and normal ageing. Our results revealed five main findings: (i) we confirmed the left-sided involvement of the hippocampus proper in the retrieval of autobiographical memories, for both recent and remote periods of life, with a greater involvement of the left hippocampus for the period of young adulthood. (ii) A lateralized pattern in hippocampal engagement with a prominent left-sided involvement for remote period and right-sided for intermediate periods. (iii) A different pattern along the rostrocaudal axis: deficit in retrieving memories from the young adulthood period was correlated with atrophy in the anterior area, while only the posterior area was correlated to more recent memory deficit. (iv) We also found other crucial regions of AbM, namely BA7, BA19, BA21, BA 25, BA47 –limited by the topography of cerebral atrophy in our patients – which confirms that our results are reliable in regard to the literature on AbM.

### 4.1. AbM behavioral results

When considering the post-hoc analyses performed on the MCT scores in the patient group, the significant group-period interaction and intra-group post-hoc analysis suggests the existence of a temporal gradient within the AD patient group with more impaired memories related to shorter retention intervals, in agreement with most of the studies on AbM in AD patients [25], [26], [27], [28], [31], [32], [33], [34], [49], [50]. However, the inter-group comparisons confirmed the AbM impairment in AD patient as compared to normal ageing for all period of life but the ‘0–9 years’ period, i.e. for extensive retention interval. This suggests that the group-period interaction might result from lower scores on the MCT in both groups for the first period rather than from a distinct temporal pattern within the two groups.

The lower scores for the childhood period was previously described during normal ageing as “childhood" amnesia [51], [52], and is hypothesized to reflect absence of encoding in an episodic mode due, among other factors, to the late maturation of the prefrontal regions' [53], [54], [55]. The lack of significant differences between the patients and controls' scores for the first period probably accounts for the absence of correlation in VBM.

Conversely, both patients and controls produced higher AbM scores for the ‘10–29 years’ period, which is consistent with the ‘reminiscence bump’ described in previous studies [51], [52]. Memories from young adulthood would be over-represented and more vivid because they are ‘self-defining’ and are considered meaningful for one's lifetime identity [4], [56].

The ‘previous year’ diminished performances in the AD patients likely results from an encoding and/or consolidation deficit secondary to hippocampal dysfunction, which was well-documented with respect to anterograde memory in AD (e.g. [57]), besides a shorter time interval during this period.

Overall, the existence of a temporal gradient in our behavioral data does not foresee the absence of hippocampal involvement in AbMs retrieval deficit for remote periods, as attested by our VBM data (see § 4.2.).

### 4.2. MTL and remoteness of the memories

The present study confirms the involvement of the hippocampus in retrieval of AbMs for both recent and remote encoding periods with the scores on the MCT (i.e. ‘10–29 years’, ‘30–59 years’, ‘after 60 years’, and ‘previous year’), during retention intervals greater than 50 years. This was previously reported in several functional imaging studies [5], [6], [8], [13], [14], [15], [16], [17], [18], [19], but only a few of them involved older subjects [7], [8], [17] – as it is the case in our study -, and thus provide an opportunity to assess the effect of the very long term retention interval. Our results, as the mentioned studies, seem to support the MTT [2], [11] and the more recent ‘transformation’ theory [12]. Moreover, similarly to Gilboa et al.'s study [58], we found a larger hippocampal involvement on the left side for the ‘10 to 29 years’ period (225 voxels), compared to the following periods (27, 90 and 17 voxels). These results also fit well with the prediction of the MTT [2], [11], where a multiplication of mnemonic traces is supposed to occur as time goes by and memories are repeated, leading to a wider distribution within the hippocampus. Nevertheless, it was not the case when comparing the ‘30 to 59 years’ (27 voxels) and ‘after 60 years’ periods (90 voxels) on the left side, or when considering the right-sided hippocampal aspects (65 voxels for the ‘10 to 29 years’ period, 50 voxels for the ‘30 to 59 years’ period, 146 voxels for the ‘after 60 years’ period) (see § 4.3. & 4.4. for detailed discussion on these issues). Additionally, this larger pattern could also be specific to the age of the person at the time of encoding rather than the duration of the retention in both Gilboa et al.'s study [58] and ours, and stand as a neuro-anatomical correlate of the ‘reminiscence bump’ ([52] ; see § 4.1.).

To date, only one study [35] has investigated the relationship between retrograde memory deficit and focal atrophy in AD. That study provided solid data in favor of the time dependency of AbM retrieval on MTL, confirming the correlation between AbM deficit and the extend of the MTL atrophy, but not with the hippocampus proper. Our findings nicely complete these previous results by showing that the hippocampus proper is involved, in addition to parahippocampal structures that are part of the ‘core network’ of AbM, namely BA 36/37 for the ‘10 to 29 years’ and ‘30 to 59 years’ periods, and BA 36 for the ‘after 60 years’ period [10]. Conversely, our results seem contradictory to a previous study which used VBM to compare AD patients' hypometabolism in resting state PET and impairment in AbM retrieval [36]. However, in this study, the lack of correlation between hippocampal hypometabolism and AbM scores for the most remote periods of life likely reflects a transition from episodic memories to ‘semanticized’ memories (see § 1.3.).

### 4.3. Lateralization in hippocampal engagement

Despite the correlations observed between the MCT and bilateral hippocampal atrophy, additional LI analyses indicated a prevailing left-sided hippocampal involvement for the ‘10–29 years’ period, and prominently right-sided in its posterior part for the intermediate periods. Together with the broader involvement of the right hippocampus for the ‘after 60 years’ period as compared to the two previous periods (146, 50 and 65 voxels respectively, see Fig. 4), this suggests a disengagement of this structure with the remoteness of the memories.

As far as AbM is concerned, bilateral activations, though predominant on the left, were found in several functional imaging studies [7], [8], [13], [14], [17], [22], [59], [60], whereas other studies demonstrated hippocampal engagement restricted on the left [15], [59] or restricted on the right [61], [62], [63]. The inconsistencies observed in MTL lateralization across AbM studies could not be readily related to the modality of the stimulus presentation, i.e. verbal or visual material used to elicit the AbMs (e.g. [64], see [65] for a review). Along these lines, two studies of patients with unilateral temporal lobectomy [66] and healthy subjects tested for their contextual memory of ‘lifelike’ events [67] help to dissociate the hippocampal functions depending on the side, with the left hippocampus engaged by retrieval of contextual details and the right by spatial memory and navigation (see [68] for a review). Moreover, Viard et al's AbM study [8] explained right hippocampal activation by the richness of the memories in terms of visuo-spatial details in healthy elderly subjects, which also seems to favor the role of the right hippocampus in visuo-spatial processing of AbMs even when no visual material is used to elicit the memories.

The bilateral hippocampal engagement was suggested as a compensatory mechanism during ageing by Maguire & Frith [14] when comparing a group of young and elderly adults which performed similarly. The additional recruitment of the right hippocampus was supposed to respond to physiological changes in the ageing brain. However in a further study [7], the authors found bilateral hippocampal activation, both in young and elderly participants. Similarly to Viard et al.'s [8], this study revealed a pattern of decreasing activity of the right hippocampus with the remoteness of the memories. In line with these two latter studies, we suggest that the different right hippocampal engagement is related to the effect of memory remoteness, rather than to the age of the subject.

The prevailing right hippocampal involvement that we found for the intermediate periods, together with parieto-occipital regions (BA 7 and BA 19), seems coherent with the notion of visuo-spatial context processed by the right hippocampus [8]. Studying the detailed phenomenological characteristics of the recollection might have revealed more visuo-spatial details for the intermediate periods in our participants, but the MCT is not well-designed as relates to strictly quantifying the contextual details.

### 4.4. Pattern of activation along the rostrocaudal axis

When considering the distribution of the left hippocampal engagement along the rostrocaudal axis and across the periods of life, we found that the hippocampus was involved in AbM retrieval in its anterior part on the left side only for the ‘10–29 years’ period, in addition to its posterior part bilaterally, as it was the case for the three following periods (see Fig. 4). Moreover, the LI adapted to analyze the relative contribution of the anterior *vs* posterior hippocampal sub-regions, confirmed that the implication of the left-anterior area was specific for the period ‘10–29 years’. We therefore suggest -with caution due to the small size of the sample- that a rostrocaudal gradient exists in hippocampal involvement during AbM retrieval depending on the encoding period. Gilboa et al.'s [6] found a reversed pattern in their functional imaging study, with the anterior areas of the hippocampus activated during retrieval of recent AbMs, whereas posterior areas activated for remote AbMs. Most importantly, this was not the case when the authors took the vividness of the memory into account, or in Viard et al.'s study [8], where no differential antero-posterior activation was detected in the hippocampus according to memory remoteness.

Additionally, our analyses showed a ‘disengagement’ of the hippocampus -in terms of volume- according to the memory remoteness for the three recent periods, with a more restricted bilateral pattern for the period ‘30–59 years’ than for the period ‘after 60 years’ (see Fig. 4). This observation seems in contradiction with the prediction of the MTT as far as the *retrieval* of AbMs is concerned. However, a simultaneous effect of encoding and/or consolidation deficit could reconcile this finding with the MTT. Considering that the pathological processes precede the onset of the deficit in anterograde memory for decades in AD [69], we suggest that an encoding and/or consolidation deficit of a complex material such as autobiographical events in the AD patients could exist years to decades before the onset of the anterograde memory deficit. The hippocampal ‘disengagement’ observed from the period ‘after 60 years’ to the period ‘30–59 years’ could therefore result from an encoding and/or consolidation deficit additional to a retrieval deficit in our patients. This is concordant with the posterior hippocampal aspects associated to AbM deficit from the ‘recent year’ which is supposed to reflect mainly an encoding deficit. This would be well accounted for by Moser & Moser's model [70] that predicts involvement of the posterior part of the hippocampus for recent visuo-spatial memory encoding in animals. Similar results were demonstrated for verbal memory [71] and for visual memory tasks [72] in humans (see also [73], [74]). This posterior aspect is also concordant with a VBM study in AD, during which the participants were assessed with a navigation task involving complex and recent visuo-spatial learning and which performances were correlated with atrophy in the right posterior hippocampus [75]. Conversely, another VBM study in AD [76] which tested the encoding of recently learned verbal material, demonstrated the implication of the anterior part of the hippocampus.

Our results suggest a *predominant* rather than *exclusive* involvement of the mentioned hippocampal sub-regions. However, our interpretations must be taken cautiously due to our small sample size, and the need of pooling our two groups of participants within the VBM analyses. Moreover the patient group was medicated heterogeneously. Finally, we were not able to perform statistical comparison between the different periods of life, which constitutes one of the main limitations of the VBM method.

### 4.5. Contribution of extra-MTL structures

Besides the MTL, the volumetry data showed that the retrieval of AbMs involves a network comprising regions which were previously implicated in autobiographical recollection (see [10] for a review), within the regions atrophied in early stages of AD [77].

The external temporal cortex (BA 20, 21, 22), and particularly the middle temporal gyrus (BA 21), which appeared for the second and third period, is known to contain the semantic component associated to episodic AbMs (e.g. [5], [14], [35], [61]). As such, this additional lateral temporal and hippocampal pattern for the remote periods could be accounted for by the “transformation" theory [12], which predicts that strictly episodic memories remain dependent on the hippocampus and that a schematic version with fewer contextual details would develop in the neocortex as time goes by.

Among the other regions described in previous studies, the ventro-lateral prefrontal cortex (BA 47) is thought to be involved in retrieval strategies and reconstructive mnemonic processes (e.g. [5], [6], [14], [78], [79]). In the same cluster, the ventro-medial prefrontal aspects (BA 25 but not the ‘core’ BA 10 in our study) is well known to be crucial for self referential processing (e.g. [6], [14], [21], [78], [79]). The orbitofrontal cortex (BA 11) was also part of the same cluster and is involved in emotional processing among other functions (see [80] for a review), particularly during memory tasks [81]. The amygdala and the insula (BA 13) are less frequently described (e.g. [7], [58], [61]) but might be associated with emotional processing, particularly with visceral representation for insula [82] and emotional aspects recollection for amygdala (see [83] for a review).The precuneus (BA 7 ; e.g. [6], [21]) and the occipital cortex (BA 19 and BA 18 ; e.g. [5], [6], [9], [60], [78]) would contain the visuo-spatial context of events. Finally, the anterior cingulate (BA 25) and the retrosplenial cortex (BA 30) are connected to each other and bind together the MTL to the anterior and posterior regions (e.g. [6], [78]).

The fact that some of the ‘core regions’ frequently involved in AbM retrieval such as BA 10 (see [10] for a review) were not revealed by our study is explained by the absence of significant atrophy in those regions in the patient group compared to the controls (results not shown), due to the early stages of the disease.

## Conclusion

Despite the relatively small number of subjects, our results could be useful since they comprise some new and complementary opening in the investigation of episodic autobiographical memory. We have confirmed the involvement of the hippocampus in AbM retrieval for both recent and remote encoding periods, with larger left-sided hippocampal aspects for the most remote period, in accordance with the MTT and the transformation theory. This study also highlights a lateralized pattern in hippocampal involvement, prominently left sided for the young adulthood period, while a right-sided hippocampal involvement prevails for more recent periods of life, which decreases with the remoteness of the memories and could be related to the visuo-spatial processing of the memories. Finally, a rostrocaudal gradient appears according to the retention duration, with anterior aspects specifically related to retrieval deficit of remote memories and posterior related to simultaneous encoding and/or consolidation and retrieval deficit of recent memories. Additionally, this study appears to be reliable as it revealed many of the regions thought to be crucial for AbM.
